# Suppression of Cellular Proliferation in PC3 Prostate Cancer Cells by Green Tea Extract Through Induction of miR‐34a Expression

**DOI:** 10.1002/fsn3.70215

**Published:** 2025-05-06

**Authors:** Seyedeh Fatemeh Soghrati Salek Moalemi, Fatemeh Safari, Hiva Ahvati

**Affiliations:** ^1^ Department of Biology, Faculty of Science University of Guilan Rasht Iran; ^2^ School of Biology, College of Science University of Tehran Tehran Iran

**Keywords:** cell proliferation, green tea extract, MiR‐34a, PC3 prostate cancer cells

## Abstract

Prostate cancer (PC) ranks as the fifth major cause of cancer‐related fatalities globally. Exploring new methods with high efficacy and low side effects by using new compounds is always desired. Tea is considered the second most commonly consumed beverage among the population of the world. Polyphenols (or catechins) in green tea play a significant role in cellular signaling pathways. Herein, we evaluate the effects of green tea extract on suppression of cellular proliferation through testing the expression of miR‐34a in PC3 prostate cancer cells. In this respect, PC3 prostate cancer cells were cultured and treated with green tea extract for 48 h. By using the qRT‐PCR method, the expression of miR‐34a was analyzed. Moreover, the expression of key proteins to regulate cellular proliferation, such as prostate specific antigen (PSA), AKT, cyclin dependent kinase 1 (CDK1), cyclin B1, c‐Myc, p53, and phospho‐androgen receptor (p‐AR) was evaluated by using western blot. Our results indicated the induction of miR‐34a, p53, and the inhibition of cyclin B1, p‐AR, CDK1, p‐AKT, PSA, c‐Myc, and p‐CDK1. Our findings can be used to design anti‐tumor regimens that utilize natural product ingredients. However, additional research will be needed to identify anticancer activities of green tea via miR‐34a in prostate cancer cells.

## Graphical abstract

Herein, we evaluated the effects of green tea extract on the suppression of cellular proliferation through testing the expression of miR‐34a in PC3 prostate cancer cells to regulate cell signaling.

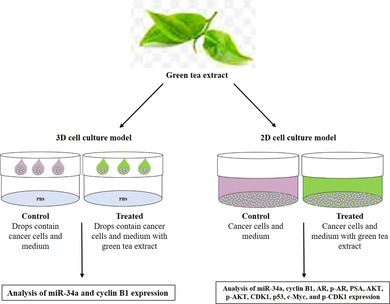

## 1 Introduction

Prostate cancer (PC) ranks as the fifth major cause of cancer‐related fatalities globally (Sung et al. 2021). Current approaches to cancer treatment are not effective, and thereby, exploring new methods with high efficacy and low side effects is always desired. Tea is the second most commonly consumed beverage among the population of the world, which has been obtained from the leaf of *Camellia sinensis*. Based on the processing or harvesting leaves development, the three tea types that are commonly used are green, black, and oolong. Green tea has a complex composition and contains different compounds such as protein, amino acids, fiber, and especially polyphenols (PPs), the most of which in green tea are flavonols. Flavonols or catechins are dihydroflavonols that are 3‐hydroxy derivatives of flavanone. Green tea products have the greatest levels of catechins compared to black or oolong tea products (Nur et al. 2021). Essentially, green tea contains different types of catechins. Green tea polyphenols (GTPs) have been demonstrated to play a significant role in cell growth and proliferation, metastasis and invasion, apoptosis, as well as angiogenesis (Hayat et al. 2015; Chacko et al. 2010; Singh et al. 2011; Tsao et al. 2009; Alavi and Safari 2025). Notably, it has been revealed that overexpression of androgen and its receptor can play a crucial role in various tumor progress and development. In this regard, it was reported that epigallocatechin gallate (EGCG) can inhibit expression levels of androgen receptor (AR) and nuclear translocation in a tumor xenograft model (Siddiqui et al. 2011). Moreover, it was shown that EGCG can modulate expression levels of PSA and AR transcription activity in LNCaP prostate cancer cells (Chuu et al. 2009; Ren et al. 2000).

MicroRNAs (miRNAs) are small and single‐stranded noncoding RNAs. They are effective at the post‐transcription level (Lee et al. 1993). MiR‐34a is encoded on chromosome 1 in the human genome, and the promoter region of miR‐34a contains a prominent CpG island and p53‐binding sites and thereby, regulation of miR‐34a is dependent on CpG methylation (inactivation) and p53 (activation). MiR‐34a is considered a kind of tumor inhibitory miRNA. Interestingly, it was noted the reduction of miR‐34a through inhibition of tumor growth promotion in vivo and in vitro (Liang et al. 2015; Yamamura et al. 2012). In this regard, it was found that p53 and c‐Myc have been found to target miR‐34a (Yamamura et al. 2012). Moreover, it was found that inhibition of AR is considered a potent target in prostate cancer therapy. Interaction between AR and a number of signaling or scaffold molecules plays significant roles in regulating the cell cycle, proliferation, and migration (Giovannelli et al. 2012; Giraldi et al. 2010; Leung and Sadar 2017).

In the current study, we concentrate on the evaluation of green tea extract effects on miR‐34a expression and targets of miR‐34a such as p53 and c‐Myc in PC3 prostate cancer cells. We also investigate the maximum vital goals in prostate cancer therapy such as AR, prostate specific antigen (PSA), AKT, cyclin B1, cyclin dependent kinase (CDK1), phospho‐androgen receptor (p‐AR), and phospho‐CDK1 (p‐CDK1: T161) expression. Our results showed that there was up‐regulation of miR‐34a in green tea extract treated PC3 cancer cells. Moreover, down‐regulation of cyclin B1, p‐AR, CDK1, PSA, p‐AKT, c‐Myc, and p‐CDK1 expression levels and up‐regulation of p53 in green tea extract treated PC3 cancer cells were detected.

## 2 Methods and Materials

### 2.1 Preparation and Extraction of Samples

The newly sprouted leaves from related plant species were gathered in Lahijan city, located in the Guilan province of Iran, and plant extract was provided as previously described. In summary, the young leaves were dried at room temperature for a duration of 14 days. Four grams of dried sample underwent extraction using 40 mL of distilled water at a temperatures ranging from 80°C to 120°C within an autoclave for a duration of 20 min, resulting in the initial extract (fraction I). Subsequently, the residues were subjected to extraction with 60 mL of distilled water at temperatures between 110°C and 130°C for 30 min, yielding fraction II. Following the cooling of the mixture to room temperature and subsequent filtering, the two fractions were merged, and it was frozen below −20°C (Safari et al. 2021; Safari et al. 2019; Safari and Dadeh Amirfard 2022).

### 2.2 Cellular Cultivation

Prostate cancer cell (PC3) was obtained from the Pasteur Institute in Tehran, Iran. These cells were cultured in DMEM medium, enriched with 10% fetal bovine serum (FBS; Bioidea BI201, Tehran, Iran), along with 100 μg/mL penicillin G/streptomycin and 1% L‐glutamine (Safari et al. 2021; Safari et al. 2019; Safari and Dadeh Amirfard 2022).

### 2.3 MTT Assay

MTT assay (MTT assay kit, Bio IDEA, CatNo: BI1017, Tehran, Iran) was performed based on our previous study (Safari et al. 2021; Safari et al. 2019; Safari and Dadeh Amirfard 2022).

### 2.4 RNA Extraction and qRT‐PCR

PC3 prostate cells were cultured and subsequently lysed after a 48‐h period during which they were exposed to green tea extract. Total RNA was then extracted using 500 μL of Trizol reagent, following the protocol provided by the manufacturer (Invitrogen Life Technologies, USA). MiR‐34a and universal reverse primers were obtained from Bon Yakhteh in Iran. MiR‐34a and universal reverse primers were 5'ATGGTGGCAGTGTCTTAGC‐3'; 5'‐GAGCAGGGTCCGAGGT‐3' (Safari et al. 2021; Safari et al. 2019; Safari and Dadeh Amirfard 2022).

### 2.5 Western Blot

Anti‐B‐actin (C4: sc‐47778 or 2A3: sc‐517582), anti‐AKT1/2/3 (5C10: sc‐81434), anti‐p‐AKT (Thr308) (B‐5: sc‐271966), anti‐PSA (A67‐B/E3: sc‐7316), anti‐c‐Myc (9E10): sc‐40, anti‐cyclin B1 (GNS1: sc‐245), anti‐p53 (DO‐1): sc‐126, and anti‐p‐AR (Ser308) (E‐6: sc‐377546) were purchased from Santa Cruz Biotechnology. Anti‐AR (ab133273) and anti‐CDK1 (ab131450) were provided by abcam. Anti‐p‐CDK1 (Thr161) (STJ90213) was prepared by St John's Laboratory. All antibodies served as primary antibodies in the immunoblotting process, and the conditions were used according to our previous studies (Safari et al. 2021; Safari et al. 2019; Safari and Dadeh Amirfard 2022).

### 2.6 DAPI Staining

DAPI staining assay was used to determine the changes in nuclear morphology of PC3 cancer cells after 48 h. This method was performed according to our previous studies (Safari et al. 2021; Safari et al. 2019; Safari and Dadeh Amirfard 2022).

### 2.7 Hanging Drop Technique

The hanging drop technique was utilized to establish a 3D cell culture model. After 3 days, spheroids were formed. The details of this method were according to our previous studies (Safari et al. 2021; Safari et al. 2019; Safari and Dadeh Amirfard 2022).

### 2.8 Statistical Analysis

The analysis of data was conducted utilizing SPSS version 22.0, while graphical representations were created with Graph Pad Prism 7 software. The results are presented as mean ± standard deviation (x ± s). Furthermore, each experiment was repeated three times, and comparisons between groups were made using an independent sample *t*‐test. A *p* value of less than 0.05 was deemed statistically significant (Safari et al. 2021; Safari et al. 2019; Safari and Dadeh Amirfard 2022).

## 3 Results

### 3.1 Up‐Regulation of miRNA34a in Green Tea Extract Treated PC3 Cancer Cells

**FIGURE 1:**
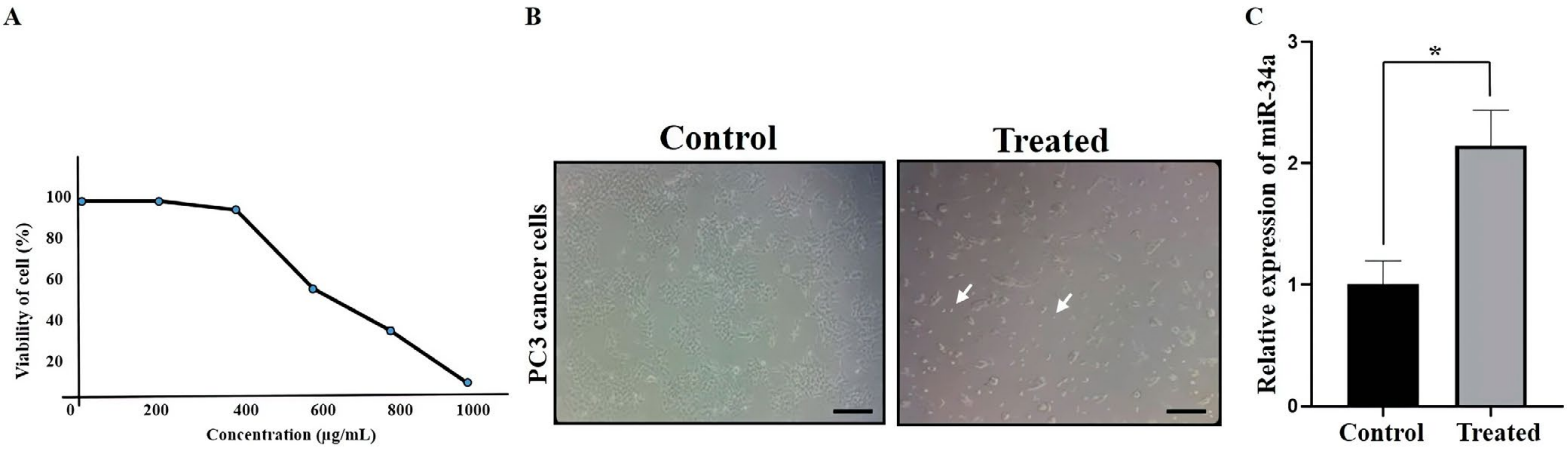
Up‐regulation of miR‐34a in PC3 cancer cells by green tea extract. PC3 cancer cell viability was shown by using MTT assay in different concentration to find IC_50_ (A); Morphological changes of PC3 cancer cells before and after treatment with green tea extract. The experiments were performed three times original microscope magnification, ×20, scale bar, 10 μm; Images were taken using a phase contrast inverted microscope (INV100, BEL Engineering, Italy). The arrows indicate cell shrinkage (B); Relative expression of miR‐34a was analyzed (C). Data represent mean ± SD of three independent experiments. *p < 0.05 was considered to be statistically significant.

To evaluate the expression of miR‐34a in green tea extract treated PC3 cancer cells, the 50% growth inhibitory concentration (IC_50_) value was detected by using the MTT assay. Therefore, the PC3 cell line was cultivated and subsequently exposed to varying concentrations of green tea extract for 48 h (Figure 1A). The IC_50_ value was 600 μM. Next, we analyzed the morphological changes of PC3 cells prior to and following treatment with green tea extract (Figure 1B). Cell shrinkage (the cells are smaller in size) was found. Following a duration of 48 h, the expression of miR‐34a was evaluated in green tea extract treated PC3 cancer cells by the qRT‐PCR method (Figure 1C). Our results indicated the induction of miR‐34a in PC3 cancer cells by green tea extract.

### 3.2 Suppression of Tumor Growth Promotion in PC3 Cancer Cells Through Inhibition of Expression of Cyclin B1, CDK1, p‐AKT, and p‐CDK1 by Green Tea Extract

**FIGURE 2:**
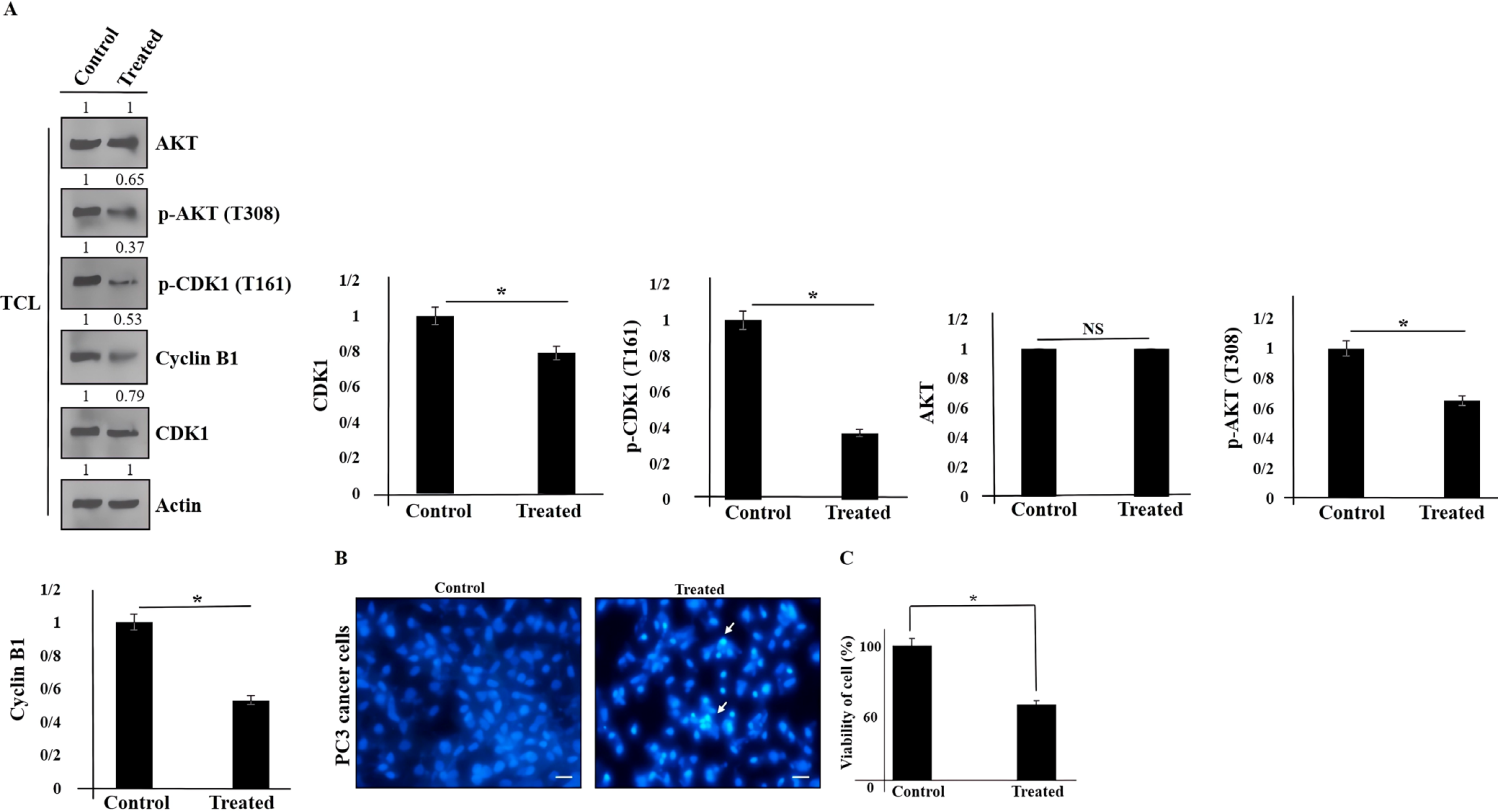
The expression of indicated proteins (cyclin B1, p‐CDK1, CDK1, AKT, and p‐AKT) versus Actin (an internal control) was shown in PC3 cells using western blot (A). (TCL: Total cell lysate). Data represent mean ± SD. *p < 0.05 was considered to be statistically significant. NS, not significant. The cellular death induction in PC3 cancer cells using DAPI staining. The experiments were performed three times (original microscope magnification, 40×, Scale bar, 10 μm; By using an inverted fluorescent microscope (Nikon Eclipse Ti‐E), all images were taken). The arrows indicate nuclear condensation of apoptotic cells (B). PC3 cancer cell viability was shown by using MTT assay (C).

It was shown that cyclin B1 and the activity of CDK1 are required to regulate the cell cycle to transit from G2 to the M phase (Gavet and Pines 2010; Tang et al. 2009). The high expression of cyclin B1 is reported in various tumors (Dong et al. 2002; Hassan et al. 2002; Patil et al. 2013; Lv et al. 2020; Song et al. 2008). Moreover, CDK1 activity requires phosphorylation at several sites including T161 (Coulonvala et al. 2011). In mitosis, AR was phosphorylated by CDK1 at the S308 site to regulate AR localization, resulting in changes in transcriptional activity (Chang et al. 1988; Koryakina et al. 2015). We found that phosphorylation of AR at the S308 site was inhibited. To find the effects of green tea extract on PC3 prostate cancer cells through inhibition of cell proliferation, we investigate the expression of cyclin B1 and CDK1 (that are important to mitosis progress) utilizing the western blot technique. We found that the expression of cyclin B1, CDK1, p‐CDK1, and p‐AKT was inhibited (Figure 2A). Then, we evaluate tumor growth promotion by using DAPI staining. We could detect condensed chromatin and DNA fragmentation of PC3 cancer cells after treatment with green tea extract. As shown in Figure 2B,C, the number of dead cells in PC3 cancer cells that were treated with green tea extract was induced.

### 3.3 Inhibition of PSA, Phosphorylated AR (At S308 Site), c‐Myc, and Up‐Regulation of p53 in PC3 Cancer Cells by Green Tea Extract

**FIGURE 3:**
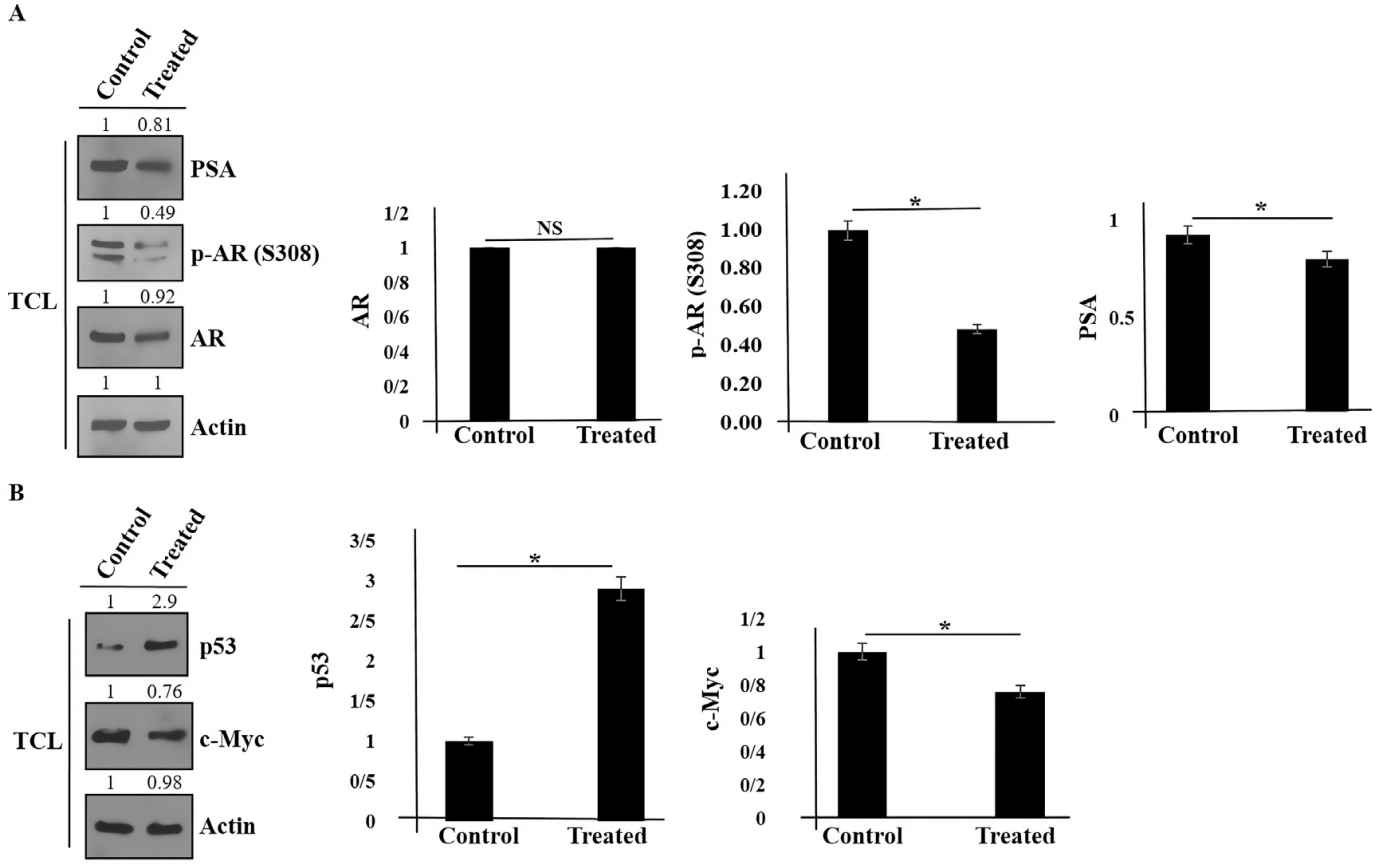
The expression of indicated proteins (AR, p‐AR, p53, c‐Myc, and PSA) versus Actin (an internal control) was shown in PC3 cells after treatment with green tea extract using western blot. (TCL: Total cell lysate) (A, B). Data represent mean ± SD. *p < 0.05 was considered to be statistically significant. NS, not significant.

The AR belongs to the nuclear receptor family, and androgens are responsible for activating AR. The role of AR in the initiation and development of prostate cancer is well established. In animal models, it was shown that the role of AR depends on its location (tumor promoter in stroma and tumor suppressor in the epithelium) (Tamburrino et al. 2012). A critical target gene for AR is PSA (Gavet and Pines 2010; Tang et al. 2009). PSA is considered a serum biomarker for the detection of PC progression. Therefore, we are interested in detecting the expression of AR and PSA in PC3 cancer cells by green tea extract. By using western blot, we found that after 48 h of treatment of androgen‐insensitive PC3 cells with green tea extract, the expression of AR did not change (Figure 3A). However, PSA expression and phosphorylation of AR at the S308 site were inhibited. It has been discovered that p53 and c‐Myc specifically target the miR‐34 family (Yamamura et al. 2012). In other words, mir‐34a suppresses prostate cancer development by cooperating with p53 and inhibiting c‐Myc. Therefore, we checked their expressions in PC3 cancer cells that were treated with green tea (Figure 3B). As shown in Figure 3B, elevation of p53 and inhibition of c‐Myc were detected.

### 3.4 Elevation of miR‐34a in PC3 Cancer Cells After Treatment With Green Tea Extract (In a 3D Cell Culture Model)

**FIGURE 4:**
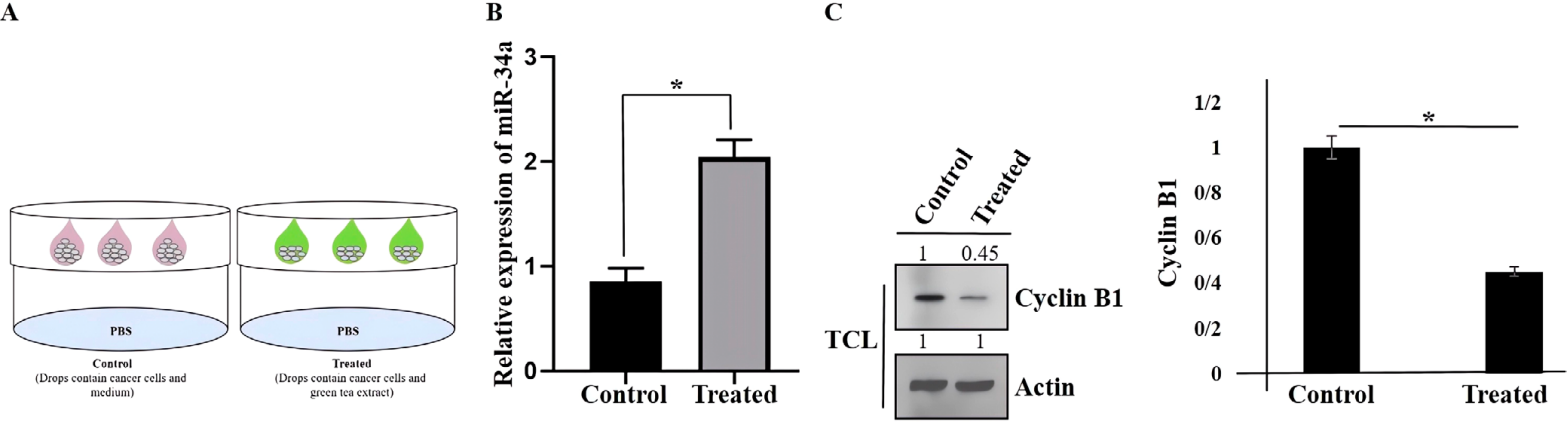
Schematic model of spheroids formation by using hanging drop technique (A). Relative expression of miR‐34a was analyzed. Data represent mean ± SD of three independent experiments. *p < 0.05 was considered to be statistically significant (B). The expression of cyclin B1 protein versus Actin (an internal control) was shown in PC3 cells using western blot. (TCL: Total cell lysate) (C). Data represent mean ± SD. *p < 0.05 was considered to be statistically significant.

Behavior of cells utilizing a 3D cell culture model is considered as in vivo cell behaviors. In order to confirm the effects of green tea extract on PC3 prostate cancer cells in a 2D cell culture model, we performed hanging drop. PC3 cells were cultured, subjected to trypsinization, and subsequently counted. Ten aliquots, each containing 20 × 10^3^ cells, were carefully pipetted into the base of the tissue culture dish lid, while PBS was added to the bottom of the dish (Figure 4A). Spheroids were formed after 3 days. Next, we analyzed the expression of miR‐34a in the treated (cells + medium containing green tea extract) compared to the control (cells + medium). As shown in Figure 4B, the expression of miR‐34a was elevated. Moreover, inhibition of cyclin B1 (as a key marker for mitosis progression) in treated cells was shown (compared control) (Figure 4C).

## 4 Discussion

Prostate cancer ranks as the fifth major cause of cancer‐related fatalities globally. Nowadays, using traditional medical strategies is considered a key way to prevent many diseases, including cancer. Cancer is a common word that is referred to as disturbing cellular pathways leading to cell proliferation through tumor formation. MiRNAs are effective at the post‐transcription level (Sung et al. 2021; Lee et al. 1993). In the current study, we focused on the evaluation of green tea extract effects on cellular proliferation through analysis of miR‐34a expression in PC3 prostate cancer cells. Our results showed that up‐regulation of miR‐34a in green tea extract‐treated PC3 cancer cells occurred. Moreover, inhibition of cell proliferation via down‐regulation of cyclin B1, p‐AR, CDK1, PSA, p‐AKT, c‐Myc, p‐CDK1 (T161) expression levels and up‐regulation of p53 in PC3 cancer cells by green tea extract was detected.

MiR‐34a is considered a potential tumor inhibitory microRNA and a therapeutic candidate in cancer (Zhang et al. 2019). Downregulation of miR‐34a was reported in various cancers such as thyroid, breast, brain, and B cell lymphoma, leading to the regulation of the expression of various genes involved in cellular proliferation and cell death (Ludvikova et al. 2015; Chen et al. 2017; Lodygin et al. 2008; Gaur et al. 2007; Fang et al. 2012). Moreover, it was indicated that the miR‐34a expression levels in prostate cancer tissues were significantly reduced compared with adjacent normal prostate tissues (Duan et al. 2015; Liang et al. 2015). Interestingly, we found that miR‐34a is elevated in PC3 prostate cancer cells by green tea extract. C‐Myc and p53 have been found to target miR‐34a (Yamamura et al. 2012). Deletion or mutation of p53 (as tumor suppressor) occurs in more than 50% of human tumors (Menendez et al. 2009). It was demonstrated that DNA damage activates the p53 gene. P53 protein binds to the promoter of miR‐34a and upregulates miRNA expression (Misso et al. 2014; He et al. 2007; Raver‐Shapira et al. 2007). Therefore, miR‐34a is a direct transcriptional target of p53. We found the upregulation of p53 in PC3 prostate cancer cells by green tea extract. The proto‐oncogene c‐Myc is frequently deregulated in various human cancers (Adhikary and Eilers 2005; Cole and Cowling 2008). In this respect, a high level of p53 and c‐Myc in prostatic carcinoma was reported (Verma et al. 2015; Fleming et al. 1986). In opposition to p53, c‐Myc represses mir‐34a transcription by binding to the conserved promoter region of mir‐34a (Chang et al. 2008), suggesting c‐Myc drives prostate cancer progression by the inhibition of mir‐34a. We showed downregulation of c‐Myc in PC3 prostate cancer cells by green tea extract. Taken together, it seems that more experiments will be required to clarify the details of the related mechanism (s).

We also found that cyclin B1 and CDK1 expression (or CDK1 activity) was inhibited by green tea extract in PC3 cancer cells. High expression of cyclin B1 accelerates mitosis to lead to excessive proliferation of cells. Dong et al. detected that cyclin B1 elevation may be related to the malignant development of laryngeal squamous cell carcinoma (Dong et al. 2002). Hassan et al. reported that after radiotherapy in patients with head and neck squamous cell carcinoma, the expression level of cyclin B1 is to be an important indicator of the risk of recurrence and metastasis (Hassan et al. 2002). In prostate cancer, high expression of cyclin B1 was correlated with tumor aggressiveness (Ersvær et al. 2020). Notably, it was shown that CDK1 was expressed at relatively higher levels in prostate cancer tissues than in normal tissues (Huang et al. 2022). We detected that green tea extract enables the inhibition of cell proliferation through suppression of cyclin B1 and CDK1 expression (or CDK1 activity) in PC3 cancer cells.

It was reported that AKT plays critical roles in regulating cell signal pathways. Moreover, AKT activity was regulated by phosphorylation of AKT at the T308 site (Alessi et al. 1996) and the high expression level of p‐Akt (T308) has been detected in metastatic prostate cancer (Ben Jemaa et al. 2015). Interestingly, we showed that phosphorylation of AKT at the T308 site was reduced, which suggests inhibiting AKT activity by green tea extract in PC3 cancer cells. However, more experiments should be performed to clarify the related mechanisms.

It should be noted that AR expression did not change in our study. However, the expression of p‐AR (at S308 site) and PSA were reduced by green tea extract in androgen‐insensitive PC3 cancer cells. AR was phosphorylated by CDK1 at the S308 site in mitosis to regulate AR localization, resulting in changes in transcriptional activity (Chang et al. 1988; Koryakina et al. 2015). In the current study, we showed the suppression of CDK1 and p‐CDK1 (at S308 site) and thereby, it confirms the inhibition effects of green tea extract on PC3 prostate cancer cells through suppression of cell proliferation.

It is worth noting that miR‐34a was inactivated by promoter CpG methylation in different cancers (Vogt et al. 2011) and EGCG can change gene expression through epigenetic modifications such as a reduction in DNA methylation by directly and indirectly inhibiting DNA methyltransferases (DNMTs) (Supic et al. 2013; Khan and Mukhtar 2010; Brueckner and Lyko 2004). Lodygin et al. reported that CpG methylation in the miR‐34a promoter and concurrent loss of miR‐34a expression was displayed in primary prostate cancer (Lodygin et al. 2008). Therefore, further studies will be required to determine the related mechanisms involved. Furthermore, bioinformatic analysis of miR‐34a targets in various cancers identifies numerous target genes such as Jagged 1 (*JAG1*), *CD44*, SRY (sex determining region Y)‐box 4 (*SOX4*), Notch homolog 2 (*NOTCH2*), Matrix metallopeptidase 9 (*MMP9*), Interleukin 6 (*IL‐6*), and SMAD family member 4 (*SMAD4*) (Soliman et al. 2018; Tang et al. 2015; Ma et al. 2017). This information may provide an opportunity to identify a critical regulatory network for predicting the molecular mechanisms of miR‐34a in the development and progression of various cancers.

In summary, we found the induction of miR‐34a in PC3 prostate cancer cells by green tea extract. Moreover, inhibition of cellular proliferation via down‐regulation of cyclin B1, p‐AR, PSA, CDK1, p‐AKT, c‐Myc, p‐CDK1 (T161) expression levels and up‐regulation of p53 in PC3 prostate cancer cells by green tea extract was detected. The current results confirm that green tea extract may be considered a potent compound in cancer therapy strategies.

## Author Contributions

**Seyedeh Fatemeh Soghrati Salek Moalemi:** formal analysis (equal). **Fatemeh Safari:** conceptualization (equal), formal analysis (equal), investigation (equal), methodology (equal), project administration (equal), supervision (equal), visualization (equal), writing – original draft (equal), writing – review and editing (equal). **Hiva Ahvati:** formal analysis (equal).

## Conflicts of Interest

The authors declare no conflicts of interest.

## Data Availability

The datasets used and/or analyzed during the current study are available from the corresponding author upon reasonable request.
